# Ag fiber/IZO Composite Electrodes: Improved Chemical and Thermal Stability and Uniform Light Emission in Flexible Organic Light-Emitting Diodes

**DOI:** 10.1038/s41598-018-37105-5

**Published:** 2019-01-24

**Authors:** Junhee Choi, Cheol Hwee Park, Jin Ho Kwack, Dong Jun Lee, Jae Geun Kim, Jaemyeong Choi, Bong Han Bae, Soo Jong Park, Enjung Kim, Young Wook Park, Byeong-Kwon Ju

**Affiliations:** 1 Display and Nanosystem Laboratory, School of Electrical Engineering, Korea University Seoul, 02841 Seoul, Republic of Korea; 2 Samsung Display Co., Samsung St. 181, Tangjeong-Myeon, Asan-si, Chungcheongnam-do 31454 Republic of Korea; 3 School of Mechanical and ICT Convergence Engineering, SUN MOON University, Asan-si, Chungcheongnam-do 31460 Republic of Korea

## Abstract

Electrospun metal fiber is a promising flexible transparent electrode owing to its extremely long length and facile fabrication process. However, metal-fiber electrodes have problems with chemical and thermal stability and nonuniform emission in organic light-emitting diode (OLED) at low luminance. In this study, we proposed a Ag fiber/IZO composite electrode with high stability. Ag fiber/IZO composite electrodes exhibited chemical and thermal stability. In addition, it was demonstrated that the OLED with the Ag fiber/IZO composite electrode operated stably, and the uniform emission of the OLED with metal-fiber electrodes improved by using highly conductive IZO film.

## Introduction

With increasing need for flexible optoelectronic devices, various flexible transparent electrodes have been intensively studied. Carbon nanotube, Graphene, conductive polymer, dielectric/metal/dielectric (DMD) and Ag nanowire have been investigated as flexible transparent electrodes^1–9^. Carbon-based materials and conductive polymer have a great potential due to their transparency and flexibility, but they have a higher sheet resistance than that of Indium Tin Oxide (ITO). DMD multi layers such exhibit the superior optical properties. However, dewetting of the thin metal on the dielectric layer is likely to occur, and this problem leads to a serious increase in sheet resistance. Among the candidates for flexible transparent electrodes that replaced brittle ITO, Ag nanowires are attracting attention owing to their comparable performance with ITO, which has a high transparency (~90%) and low sheet resistance (15 Ω/sq)^6–9^. However, Ag nanowires inevitably induce surface roughness owing to the junctions between the Ag nanowires when forming a Ag nanowire network. The surface roughness of the Ag nanowire induces leakage current and electrical short circuit in thin film devices.

Recently, electrospun metal fiber has offered attractive approaches due to its facile fabrication process and lower percolation threshold than metal nanowires; therefore, they have been recently applied to various electronic devices^10–14^. Moreover, junction-free electrospun metal-fiber electrodes have been applied to thin film devices, including organic solar cell and organic light-emitting diodes (OLEDs), because they can control the surface roughness, which is a serious problem of metal nanowires^15–18^.

However, metal fiber electrodes are vulnerable to post-heat and -chemical treatments that can occur after the electrode fabrication in the device fabrication process. For example, metal-fiber electrodes may be vulnerable to developing and baking processes for pixel define layer formation in the OLED manufacturing processes. In addition, an OLED with a metal-fiber electrode does not emit uniform light at low luminance because an electrical current cannot flow in the void space between the metal fibers and only flows through the metal fiber in the metal-fiber-based electrode. Even when buffer layers such as PEDOT: PSS, metal oxide, and graphene are coated on a metal wire-based electrode^19–25^, uniform light emission cannot be achieved due to their large resistance difference between the covered layer and metal fibers.

In this study, we fabricated junction-free Ag fiber/indium zinc oxide (IZO) composite electrodes by sputtering IZO on a Ag fiber. By introducing an IZO buffer layer on the Ag fiber, it is possible to obtain chemical and thermal stability. A sputtered IZO film that does not act as a planarization layer can be used as a buffer layer on a junction-free Ag fiber electrode with low surface roughness for stable OLED operation. In addition, the uniform emission of the OLED with a Ag fiber electrode can be achieved by depositing the IZO film, which has a resistance equal to that of the Ag fibers because the Ag fiber/IZO composite electrode has a parallel resistance of two materials—Ag fiber and IZO film.

## Results and Discussion

**Figure 1:**
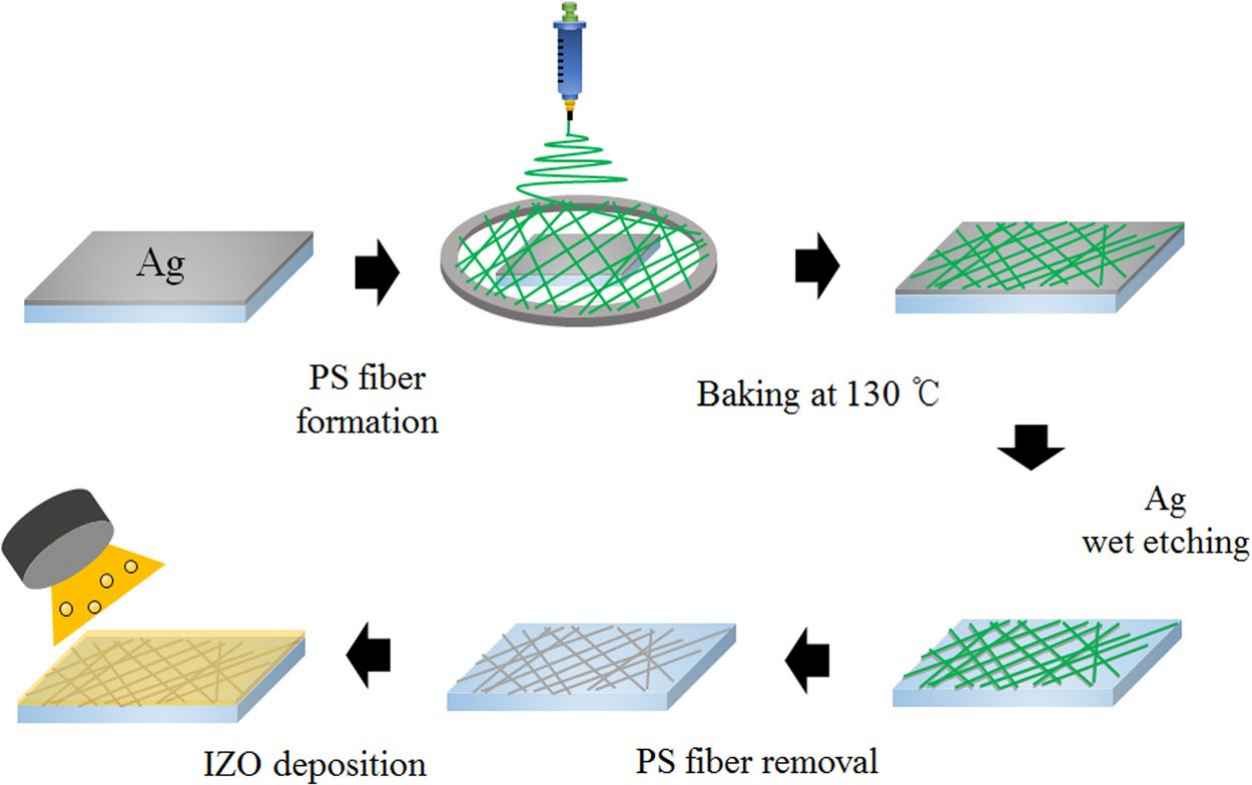
Schematic illustration of the fabrication procedure of the Ag fiber/IZO composite electrodes.

Figure 1 illustrates the entire fabrication process of the Ag fiber/IZO composite electrodes. These electrodes are fabricated by wet-etching of Ag thin films with a polystyrene (PS) fibers mask^15^ and subsequent sputtering of an IZO film on the Ag fiber electrodes. First, a polyethylene naphthalate (PEN) substrate is cleaned by UV-O treatment, and a Ag thin film with 40 nm thickness is deposited by thermal evaporation system. PS fibers are formed by the electrospinning process on the deposited Ag thin film, and the PS fibers are annealed on a hot plate at 130 °C for 10 min to improve substrate adhesion. The Ag films not covered with the PS fiber mask are etched by O_2_ plasma treatment and subsequent immersion in H_2_O_2_ solution. The PS fibers are dissolved in the chloroform solution. The IZO thin films are sputtered on the fabricated Ag fiber electrode.

**Figure 2:**
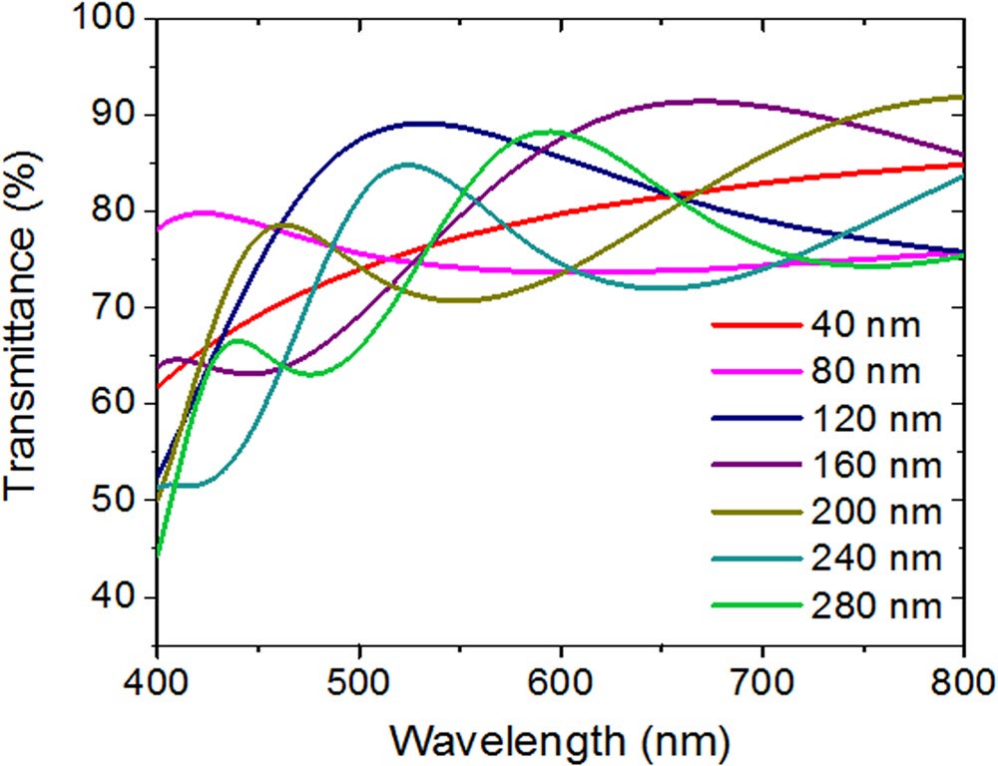
Calculated IZO thin-film transmittances at various thicknesses.

The transmittance of the IZO thin films was calculated to determine the optimum IZO thickness. Figure 2 shows the calculated transmittances by finite-difference time-domain (FDTD) optical simulator. It shows the maximum transmittance peaks depending on the IZO thickness at a specific wavelength due to Fabry–Perot interference. The IZO thickness was determined to fabricate a green fluorescent OLED with an emission wavelength of 525 nm. When the thicknesses of the IZO film are 120 nm and 240 nm, the peak transmittances are observed at a wavelength of 525 nm.

**Figure 3:**
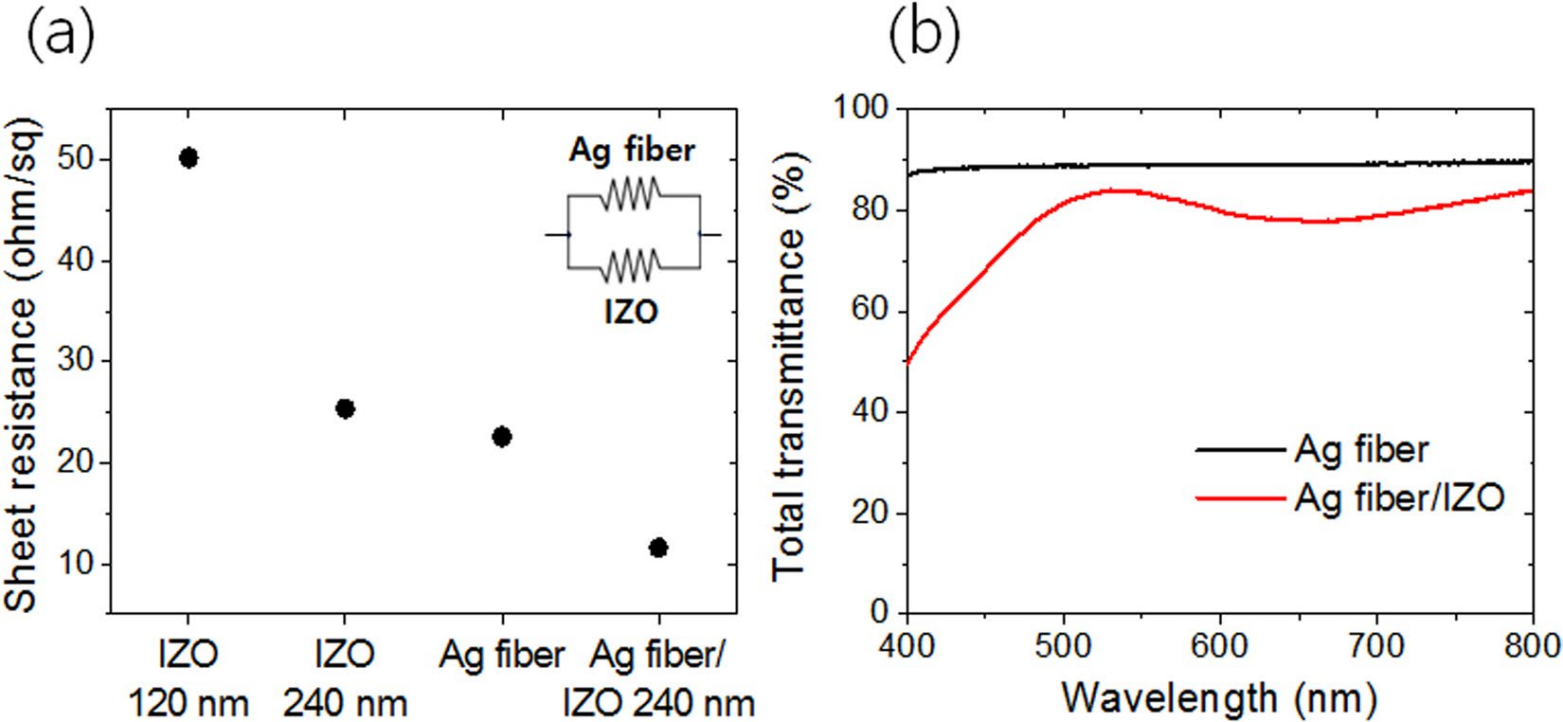
(**a**) Sheet resistances of IZO, Ag fiber, and Ag fiber/IZO composite electrodes, and (**b**) total transmittance of Ag fiber and composite electrode of Ag fiber/IZO with 240 nm thickness.

Figure 3 shows the sheet resistance and total transmittance for the IZO films and Ag fiber electrodes. The sheet resistances of the IZO films with thicknesses 120 nm and 240 nm are 50.2 Ω/sq and 25.4 Ω/sq, respectively. The sheet resistance of the Ag fibers is adjustable by spinning time and Ag thickness^15^. To obtain uniform current distribution, the resistances of the Ag fiber and IZO film should be the same because the composite electrode has a parallel current path^26–29^. The inset in Fig. 3 shows the parallel resistor circuit diagram of the Ag fiber and IZO composite electrode. Therefore, we fabricated an Ag fiber electrode with a resistance of 22.6 Ω/sq, which is almost the same as the sheet resistance the IZO film of 240 nm. The sheet resistance of the Ag fiber/IZO 240 nm composite electrode is 11.6 Ω/sq, which is comparable to the conventional ITO resistance. The composite electrode has better electrode properties than a single IZO film due to Ag fibers having good electrical and optical properties.

Figure 3(b) shows the total transmittances of the Ag fiber and composite electrodes. The transmittances were measured by UV-Vis spectrometer. The baseline is the PEN substrate. The transmittance of the Ag fiber electrode is approximately 90% in a broad-range wavelength. On the other hand, the Ag fiber/IZO composite electrode shows a peak transmittance of 83.6% at 525 nm (green). This can be explained by the Fabry–Perot interference of the IZO thin film as well as the simulation results.

**Figure 4:**
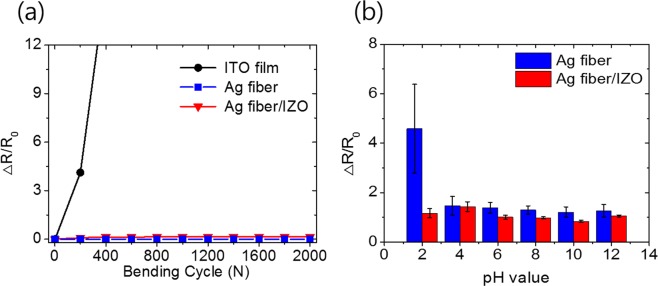
(**a**) Resistance changes in the cyclic bending test and (**b**) immersed in different pH solutions for chemical stability.

To investigate mechanical durability, a repetitive bending test was performed. The bending radius was 5 mm, and the bending cycles were 2000 times. Figure 4(a) shows the resistance changes of different electrodes under a repetitive bending test. The resistance of the ITO film increases sharply as the bending cycle increases. On the other hand, the resistances of the Ag fiber electrode and Ag fiber/IZO composite electrode did not increase depending on the bending cycle. It was observed that the Ag fiber/IZO composite electrode had mechanical durability. To investigate the chemical stability, the Ag fiber and Ag fiber/IZO electrodes were immersed in different pH solutions for 20 min. Figure 4(b) shows the resistance change of the electrode depending on different pH solutions. The resistance of the Ag fiber electrode shows no distinct changes while immersing in solutions with pH 4–12. In the case of solutions with pH 2, the resistance change of the Ag fiber electrode is approximately 4.5. On the other hand, the resistance of the Ag fiber/IZO composite electrode hardly increases because the IZO film acts as a protection layer for the chemically vulnerable Ag fiber. Therefore, the Ag fiber/IZO composite electrodes show superior chemical stability.

**Figure 5:**
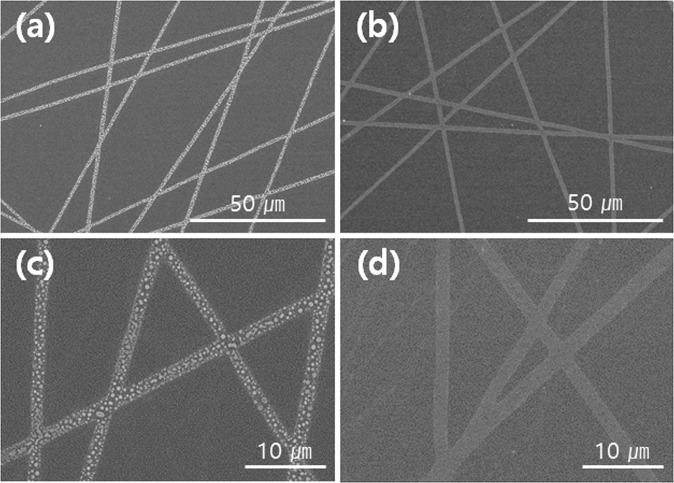
SEM images of the Ag fiber at magnifications of (**a**) × 1000, (**c**) × 3000, and Ag fiber/IZO at magnifications of (**b**) × 1000 (**d**) × 3000 after thermal annealing.

To investigate the thermal durability of the Ag fiber/IZO composite electrodes, we fabricated the Ag fiber and Ag fiber/IZO composite electrodes on the polyimide substrate, and then, the electrodes were annealed by using the rapid thermal process system at 400 °C for 10 min in air. The sheet resistances of the electrodes were measured before and after annealing, and Table 1 presents the sheet resistances. The surface morphology of the Ag fiber and Ag fiber/IZO composite electrodes are measured by the scanning electron microscope (SEM). Figure 5(a–d) show the SEM images of the Ag fiber and Ag fiber/IZO electrodes after the annealing process. Ag is generally dewetted in the form of particles by the annealing process^30,31^. In the case of the Ag fiber without the IZO layer, the Ag fiber was changed into Ag islands. (Fig. 5a,c) The network of Ag fibers was broken; thus, the resistance cannot be measured. On the other hand, the Ag fiber/IZO composite electrode shows remarkable stability. (Fig. 5b,d) The Ag fibers were not damaged at all due to the IZO buffer layer. However, the resistance of the composite electrode increased slightly after the heat treatment because the oxygen vacancy decreased by the oxidation of the IZO film, and the resistivity of the IZO film increased^32,33^.

**Table 1:** Sheet resistance of Ag fiber and Ag fiber/IZO composite electrodes before and after annealing.

Electrode Type	Before Annealing	After Annealing (400 °C)
Ag fiber	22.6	—
Ag fiber/IZO	11.6	15.5

**Figure 6:**
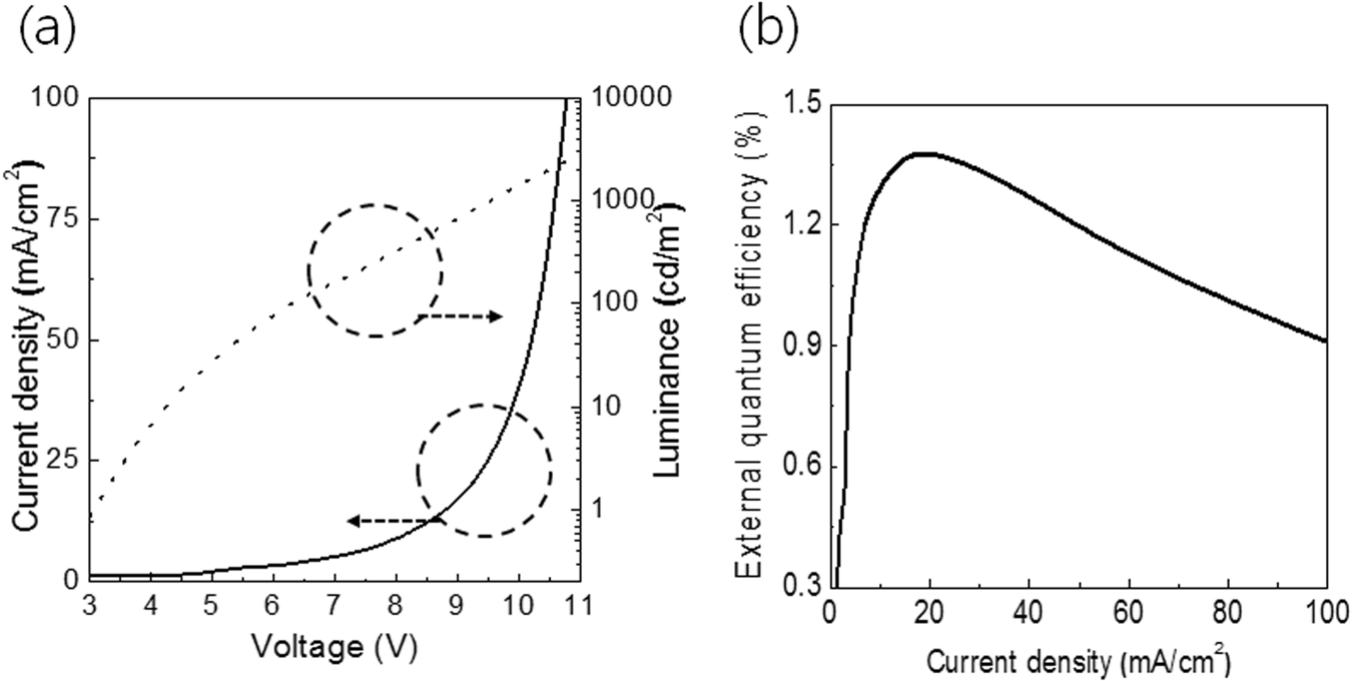
EL characteristics of OLED with the Ag fiber/IZO composite electrodes. (**a**) J-V-L characteristics, (**b**) external quantum efficiency.

To demonstrate the possibility of using Ag fiber/IZO composite electrode with a flexible OLED, we fabricated a flexible fluorescent OLED with the Ag fiber/IZO composite electrode. Figure 6(a) presents the J-V-L characteristics of the fabricated OLED. The OLED with the Ag fiber/IZO composite electrode showed stable operation without leakage current, and the turn-on voltage was 3 V. Figure 6(b) exhibits the external quantum efficiency of the OLED with the Ag fiber/IZO composite electrode. The maximum external quantum efficiency is 1.39%. It is the general efficiency of fluorescent NPB/Alq3 based OLEDs^34–37^.

**Figure 7:**
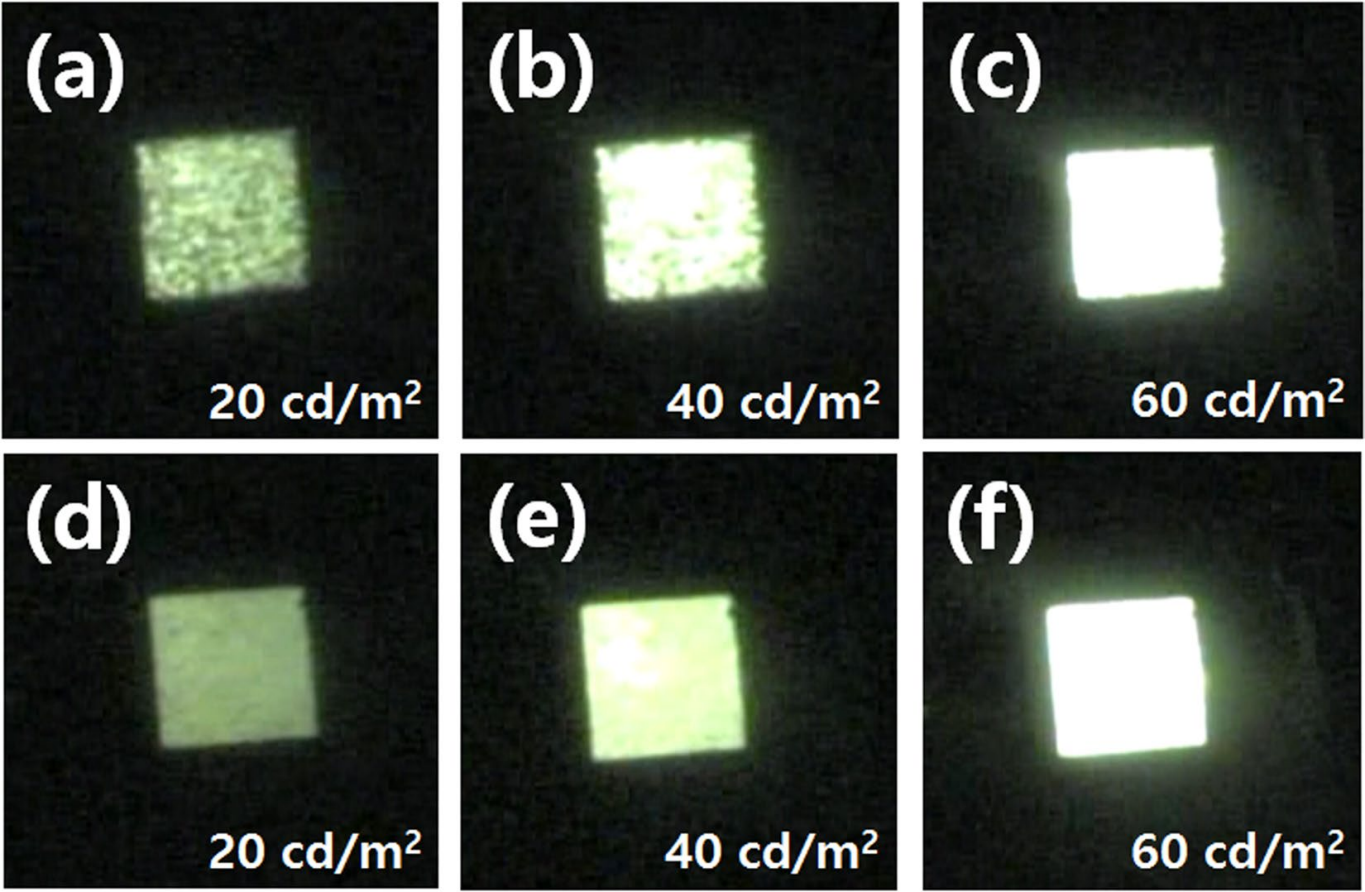
Photographs of the OLED emissions with the Ag fiber electrode under (**a**,**b**,**c**) PEDOT:PSS buffer layer and (**d**,**e**,**f**) IZO at different luminances.

Figure 7 shows the photographs of the OLEDs with different buffer layers and different luminances. Figure 7(a–c) shows the OLEDs with the PEDOT:PSS buffer layer on the Ag fibers, and Fig. 7(d–f) shows the OLED with the IZO buffer layer on the Ag fiber at luminances of 20 cd/m^2^, 40 cd/m^2^, and 60 cd/m^2^, respectively. When the PEDOT: PSS buffer layer is applied, the resistance of PEDOT: PSS is larger than that of the Ag fiber. Therefore, it was observed that nonuniform emission occurs at low luminances of 20 cd/m^2^ and 40 cd/m^2^. When the luminance was 60 cd/m^2^, the light emission became uniform. On the other hand, when the IZO buffer layer was applied, it was observed that the uniform emission was maintained at a low luminance of 20 cd/m^2^ by controlling the IZO film resistance to be the same as the resistance of the Ag fibers.

In summary, we demonstrated a Ag fiber/IZO composite electrode with chemical and thermal stability and observed uniform emission of an OLED with the Ag fiber/IZO composite electrode. FDTD optical simulation was performed to observe the optimal IZO thickness. A sputtered IZO buffer layer protected the Ag fibers from chemical and thermal damages and supported OLED uniform emission by equaling the resistance to that of the Ag fiber, which was possible because the IZO films had high conductivity.

## Methods

### Preparation of Ag Fiber/IZO Composite Electrodes

PEN substrates were cleaned by UV-O treatment for 10 min. Ag thin film with 40 nm thickness were deposited on PEN substrate by a thermal evaporator. Polystyrene powder with molecular weight 192,000 g/mol was purchased from Sigma-Aldrich Korea Ltd. Polystyrene solution is prepared by mixing 400 mg of polystyrene into 2 ml of acetone and 1 ml of dimethylformamide. The polystyrene fiber were deposited on Ag thin films by electrospinning process at 6 kV with an injection rate of 20 μL min^−1^. The electrospun polystyrene fibers were annealed using a hot plate at 130 °C for 10 min to promote the adhesion between the fibers and substrate. After heat treatment, the O_2_ plasma was treated to oxidize Ag thin film not covered by the Ppolystyrene fibers at 100 W for 5 min under a pressure of 500 mTorr. The samples were immersed in H_2_O_2_ solution for 10 s to remove the oxidized Ag film. Next, the samples were immersed in chloroform for 5 min to remove the polystyrene fibers. Finally, the indium zinc oxide films were deposited on fabricated Ag fiber electrode.

### Fabrication of Flexible OLEDs

The organic layers and cathode were deposited using a thermal evaporator (Digital Optics Vacuum Co., Ltd.) under high vacuum (10^−6^ Torr). To compare the uniform light emission, the IZO and PEDOT:PSS buffer layers were deposited. The PEDOT:PSS (Clevios PH1000 from Heraeus) buffer layers were deposited by spin-coating on Ag fiber electrodes at 1000 rpm for 30 s, then was baked at at 150 ^o^C for 10 min. The fluorescent green OLED consisted of 5 nm hexaazatriphenylenehexacarbonitrile (HATCN)/60 nm N, N′-bis (naphthalen-1-yl)-N, N′-bis (phenyl)-benzidine (NPB)/80-nm-thick tris (8-hydroxy-quinolinato) aluminum (Alq3). The HATCN, NPB, and Alq3 acted as hole injection layer, hole transport layer, and emission layer, respectively. A 0.7-nm-thick lithium fluoride (LiF) and 100-nm-thick Al was deposited as cathode.

### Characterization

The transmittances were measured by a Cary 5000 (Varian/Agilent) UV–visible spectrometer. The sheet resistances were analyzed by a standard 4-point measurement. The mechanical durability test was performed using a bending tester (Z-Tec. Co., Inc.) To investigate the chemical stability, the pH solutions were prepared by mixing an HCl and NaOH solution with Di water. To observe the thermal stability, the surface morphology of the Ag fiber and the composite electrode were measured using a field emission scanning electron microscope (S-4800, Hitachi, Ltd.). The electroluminescence characteristics of the OLEDs were measured using a source-measurement unit (Model 237, Keithley Instruments, Inc.) and a spectroradiometer (PR-670 SpectraScan, Photo Research, Inc.).
